# The *Neisseria gonorrhoeae* Methionine Sulfoxide Reductase (MsrA/B) Is a Surface Exposed, Immunogenic, Vaccine Candidate

**DOI:** 10.3389/fimmu.2019.00137

**Published:** 2019-02-06

**Authors:** Freda E.-C. Jen, Evgeny A. Semchenko, Christopher J. Day, Kate L. Seib, Michael P. Jennings

**Affiliations:** Institute for Glycomics, Griffith University, Gold Coast, QLD, Australia

**Keywords:** methionine sulfoxide reductase, bactericidal activity, opsonophagocytic activity, *Neisseria gonorrhoeae*, gonococcus, vaccine

## Abstract

Control of the sexually transmitted infection gonorrhea is a major public health challenge, due to the recent emergence of multidrug resistant strains of *Neisseria gonorrhoeae*, and there is an urgent need for novel therapies or a vaccine to prevent gonococcal disease. In this study, we evaluated the methionine sulfoxide reductase (MsrA/B) of *N. gonorrhoeae* as a potential vaccine candidate, in terms of its expression, sequence conservation, localization, immunogenicity, and the functional activity of antibodies raised to it. Gonococcal MsrA/B has previously been shown to reduce methionine sulfoxide [Met(O)] to methionine (Met) in oxidized proteins and protect against oxidative stress. Here we have shown that the gene encoding MsrA/B is present, highly conserved, and expressed in all *N. gonorrhoeae* strains investigated, and we determined that MsrA/B is surface is exposed on *N. gonorrhoeae*. Recombinant MsrA/B is immunogenic, and mice immunized with MsrA/B and either aluminum hydroxide gel adjuvant or Freund's adjuvant generated a humoral immune response, with predominantly IgG1 antibodies. Higher titers of IgG2a, IgG2b, and IgG3 were detected in mice immunized with MsrA/B-Freund's adjuvant compared to MsrA/B-aluminum hydroxide adjuvant, while IgM titers were similar for both adjuvants. Antibodies generated by MsrA/B-Freund's in mice mediated bacterial killing via both serum bactericidal activity and opsonophagocytic activity. Anti-MsrA/B was also able to functionally block the activity of MsrA/B by inhibiting binding to its substrate, Met(O). We propose that recombinant MsrA/B is a promising vaccine antigen for *N. gonorrhoeae*.

## Introduction

*Neisseria gonorrhoeae* is an obligate human pathogen that infects human mucosal surfaces and causes the sexually transmitted infection gonorrhea. It is estimated that there are more than 106 million cases of gonorrhea worldwide each year (1). Symptomatic gonococcal infection typically presents as urethritis in males and cervicitis in females, although infection of the rectum, pharynx, and eye also occur in both sexes (2). Furthermore, asymptomatic infections are common and can occur in up to 80% of infected females and 40% of infected males. If left untreated, gonorrhea can lead to severe sequelae, such as pelvic inflammatory disease, adverse pregnancy outcomes, neonatal complications, and infertility, and can also increase the risk of acquiring and transmitting HIV [reviewed in Edwards et al. (3)].

The recent emergence of multidrug resistant strains of *N. gonorrhoeae* has generated a major public health challenge. Cephalosporins are now the last line of defense for treating gonorrhea, however, isolates with high-level resistance to the expanded-spectrum cephalosporins, ceftriaxone and cefixime, have been identified globally (4), highlighting the requirement for novel therapeutic approaches or for a vaccine. Various potential vaccine targets have been described, however there are several challenges to developing a gonococcal vaccine. The challenges include the lack of protective immunity following infection, and the absence of a correlates of protection, as well as the high level of phase and antigenic variation of *N. gonorrhoeae* surface antigens [reviewed in Edwards et al. (3) and Rice et al. (5)]. Ideally, vaccine antigens should be conserved, immunogenic, and be able to induce functional antibodies that are able to mediate bactericidal or opsonophagocytic killing, and/or that are able to block an important function of *N. gonorrhoeae* [reviewed in Edwards et al. (3)].

Mechanisms for coping with oxidative stress are crucial for the survival of human pathogens such as *N. gonorrhoeae*, which are routinely exposed to oxidative killing by the host and are frequently isolated within polymorphonuclear leukocytes (PMNs) (6). Methionine residues in proteins can easily be oxidized by the presence of reactive oxygen species, affecting protein structure and function (7). The enzyme methionine sulfoxide reductase (Msr) can repair oxidized methionine by catalyzing the reduction of methionine sulfoxide residues [Met(O)] back to methionine (Met) in the cytoplasmic methionine pool and in damaged proteins (8). Pathogenic bacteria like *Escherichia coli* (9), *Helicobacter pylori* (10)*, Pseudomonas aeruginosa* (11)*, Streptococcus pneumoniae* (9, 12), and *Staphylococcus aureus* (13) all contain Msr enzymes that protect against oxidative damage. In *N. gonorrhoeae*, and the closely related bacterial pathogen *Neisseria meningitidis*, the Msr enzyme is a three domain protein named MsrA/B, which contains a thioredoxin-like disulfide reductase, a methionine sulfoxide reductase A and a sulfoxide reductase B (14, 15). MsrA/B of *N. gonorrhoeae* was initially named PilB and was thought to be a pili regulatory protein involved in bacterial adherence to host cells (16). However, later studies disproved this role and showed that the gonococcal MsrA/B is involved in protection from oxidative stress (17), and is upregulated by H_2_O_2_ (18) and Ecf/sigma E (19). Structural and catalytic studies of MsrA/B revealed its roles in binding and reducing both Met-R(O) and Met-S(O) epimers in *N. gonorrhoeae* (14, 20) and *N. meningitidis* (15, 21–28). The majority of Msr proteins are found in the cytoplasm, where they are collocated with a thioredoxin/thioredoxin reductase system that is involved in regenerating oxidized Msr back to its active form (29). However, based on cell fractionation experiments the gonococcal MsrA/B was proposed to be present in two forms, a truncated inactive cytoplasmic form and a full-length active form located in the outer membrane (17), and presumed to be facing the periplasm (20, 24).

In this study, we examine the conservation and localization of MsrA/B in *N. gonorrhoeae* and *N. meningitidis*. We also investigate the level, type, and functional activity of antibodies raised to the recombinant gonococcal MsrA/B antigen in order to assess its potential as a gonococcal vaccine candidate.

## Materials and Methods

### Bacterial Strains and Growth Conditions

*N. gonorrhoeae* 1291 (30), 20 clinical isolates from mucosal and disseminated gonococcal infections (31) and *N. meningitidis* MC58¢3 (32) strains were grown on GC agar (Oxoid) with 1% IsoVitaleX (Becton Dickinson) or Brain Heart Infusion (BHI, Oxoid) 1% agar with 10% Levinthal's Base medium at 37°C with 5% CO_2_, respectively, with either kanamycin (kan) (100 μg/ml) or tetracycline (5 μg/ml) as required.

### Sequence Bioinformatics Analysis

Distribution of MsrA/B in gonococcal genomes, available at GenBank and at the Meningitis Research Foundation (MRF) Meningococcus Genome Library (PubMLST) database, was investigated using BLAST search with MsrA/B from *N. gonorrhoeae* 1291 (GenBank Accession: protein-EEH61172.1; nucleotide-DS999919.1, Locus tag NGAG_00088).

### Generation of Mutant Strains

The 1,569 bp *msr* gene from *N. gonorrhoeae* 1291 was amplified with primers 1291pilBFor and 1291pilBRev (see Table S1 for primers) containing the *Neisseria* uptake sequence 5′-GCCGTCTGAA-3′ and the resulting PCR product was cloned into pGem ®-T Easy (Promega) to generate pGemT*msr*. The Mutation Generation System™ (MGS) kit (Thermo Fisher) was used according to the manufacturer's instructions to insert a transposon containing a *kanR3* gene into pGemT*msr*. The location and orientation of the *kanR3* in *msr* was determined by sequencing. The *msr::kan* construct was linearized and transformed into *N. gonorrhoeae* 1291 and *N. meningitidis* MC58¢3 by homologous recombination to generate 1291*msr::kan* and MC58¢3*msr::kan* mutant strains. To generate complemented strains, the intact *msr* gene was introduced into either the 1291 *msr::kan* mutant using the complementation plasmid pCTS32 (33), or the MC58¢3 *msr::kan* mutant using pComPind (34).

### MsrA/B Protein Expression

The *msr* gene was amplified from *N. gonorrhoeae* 1291 using primers msrexp_NdeIF and msrexp_XhoIR (Table S1) and the resulting PCR product was cloned into pET15b to obtain a Msr expression construct pET15bmsr. The construct pET15bmsr was transformed into *E. coli* BL21 Star (DE3)pLysS host strain (Novagen) and MsrA/B was overexpressed and purified. Briefly, expression was induced by 0.1 mM IPTG at an optical density at 600 nm (OD_600_) of 0.4 for 24 h at room temperature. Cell cultures were harvested and the cell pellet was re-suspended in buffer A. Cells were lysed by sonication, centrifuged, and the supernatant was applied to a column packed with TALON™ metal affinity resins (Clontech Laboratories, Inc.). Unbound proteins were washed away with 20 column volumes of buffer A, followed by 10 column volumes of buffer A with 20 mM imidazole. The MsrA/B protein was eluted in fractions of 1 ml of 200 mM imidazole. Fractions were collected and analyzed by 4–12% NuPAGE® Novex Bis-Tris Gels (Invitrogen) stained with Coomassie Blue, and by Western blot of anti-His polyclonal antisera.

### MsrA/B Mouse Antisera Production (anti-MsrA/B)

Groups of 10 female BALB/c mice (6 weeks old) were immunized subcutaneously with 5 μg of recombinant MsrA/B with either Alhydrogel® (aluminum hydroxide, InvivoGen) or Freund's (FCA/FIA, Sigma-Aldrich) adjuvant on days 0, 21, and 28. Terminal bleeds were collected on day 42. For Freund's adjuvant, Freund's complete adjuvant (FCA) was used on day 0 and Freund's incomplete adjuvant (FIA) was used in the boosts of day 21 and 28. Pre-bleed of each mouse was collected 4 days before immunization. This study was carried out in accordance with the recommendations of the Australian Code for the Care and Use of Animals for Scientific Purposes, the Griffith University Animal Ethics Committee (AEC). The protocol was approved by the Griffith University AEC.

### Cell Surface Trypsin Digestion

Overnight culture of 1291 and MC58¢3 were inoculated into appropriate media at an OD_600_ of 0.05. After 2 h growth at 37°C, cells were harvested, washed once, and resuspended in PBS to an OD_600_ of 2. Cell suspension (200 μl) were treated with trypsin (trypsin gold, Promega) for 60 min at 37°C. Cell suspensions at time 0 and at 60 min were taken in triplicate for the determination of colony forming units (CFUs)/ml to confirm cell viability, and were analyzed by Western blot analysis with anti-MsrA/B. Control antibodies used were to surface exposed PorA (NIBSC-UK-EN63QFG) and cytoplasmic GNA2091 (35, 36).

### ELISA

For whole cell ELISA, bacteria were grown on BHI or GC plates for 16 h. Cells were harvested and resuspended in PBS at an OD_600_ of 0.2. Microtiter plate wells were filled with 50 μl of the bacterial suspension and dried at room temperature overnight in the laminar flow cabinet. After the bacteria in the dried wells were heat-killed for 1 h in 56°C. For recombinant protein ELISA, wells of plates were coated with 100 ng of purified recombinant MsrA/B protein in 100 μl of coating buffer (0.5 M carbonate/bicarbonate buffer, pH 9.6) for 1 h at room temperature. All ELISAs were performed with mouse pre-immune or MsrA/B immunized sera, and secondary antibody as specified in the results [polyclonal anti-mouse Ig HRP (Dako) or IgG1, IgG2a, IgG2b, IgG3, or IgM HRP (Thermofisher Scientific)]. The substrate TMB (3,3′, 5,5;-tetramethylbenzidine) solution (Thermofisher Scientific) was used as per manufacture's instruction. Equal amount of 1 N hydrochloric acid was added to stop the reaction. Absorbance was read in a TECAN Model Infinite 200 Pro plate reader at 450 nm.

### Serum Bactericidal Assay

*N. gonorrhoeae* 1291 (~1 × 10^3^ CFU) was incubated in serial dilutions of heat-inactivated (56°C, 60 min) anti-MsrA/B sera or pre-immune sera for 15 min at 37°C, after which normal human serum [pre-absorbed with *N. gonorrhoeae* as described previously (37)] was added to a final concentration of 10% (v/v) as a source of complement. The suspension was then incubated at 37°C, 5% CO_2_ for 30 min and bacterial CFU determined by plating out serial dilutions. The bactericidal titer is the reciprocal of the lowest antibody dilution, which induced more than 50% killing after 30 min. Statistical significance was calculated using one-way analysis of variance (ANOVA) and Student's *t*-test.

Whole blood from healthy volunteers was collected by venipuncture. For serum, blood was collected in Vacuette Z serum separator tubes (Greiner Bio-One), allowed to clot for 15 min at room temperature then centrifuged for 10 min at 2,000 × *g*. This study was carried out in accordance with the recommendations of the National Statement on Ethical Conduct in Human Research, the Griffith University Human Research Ethics Committee, with written informed consent from all subjects. All subjects gave written informed consent in accordance with the Declaration of Helsinki. The protocol was approved by the Griffith University Human Research Ethics Committee.

### Opsonophagocytic Killing Assay

Polymorphonuclear leukocytes (PMNs) were isolated from donor blood [collected in K3 EDTA tubes (Greiner Bio-One)] using Polymorphprep™ (Axis-Shield) as per manufacturer's instructions, and were resuspended in assay buffer (RPMI (Gibco) supplemented with 0.15 mM CaCl_2_, 0.5 mM MgCl_2_ and 0.5% (v/v) human serum albumin). *N. gonorrhoeae* 1291 (~1 × 10^3^ CFU) was incubated in serial-dilutions of heat-inactivated anti-MsrA/B sera or pre-immune mouse sera for 15 min at 37°C. PMNs (~1 × 10^5^ cells) and a complement source (10% v/v normal human serum pre-absorbed with *N. gonorrhoeae*) were then added, and incubated at 37°C for 90 min. Gonococcal survival was determined after plating of serial dilutions on GC agar, and survival calculated as a percentage relative to no-antibody control. The opsonophagocytic titer is the reciprocal of the lowest antibody dilution, which induced more than 50% killing after 90 min. Statistical significance was calculated using one-way analysis of variance (ANOVA) and Student's *t*-test.

### Surface Plasmon Resonance (SPR)

SPR assays were performed using a Biacore T200 for affinity analysis and a Pall Pioneer FE for competition assays. Affinity assays were performed as previously described (38). Briefly, MsrA/B was immobilized onto flow cell 2 of a Series S CM5 sensor chip using amine coupling kit (GE Life Sciences) at a flow rate of 5 μL/min for 10 min. Flow cell 1 was used as the reference cell and immobilized with ethanolamine only. Met(O) was run at a final concentration range of 0.16–100 nM using single cycle kinetics. Data was analyzed using the Biacore T200 evaluation software package. For competition analysis, MsrA/B was immobilized onto flow cell 1 of a COOH5 Biosensor chip and flow cell 2 the blank immobilized surface using amine coupling EDC-NHS reactions. Briefly, EDC-NHS mix was flowed at 10 μL/min for 10 min across flow cell 1 and 2. MsrA/B was then flowed across flow cell 1 at 5 μL/min for 20 min in sodium acetate pH 4.5 at a concentration of 25 μg/mL. Ethanolamine was then flowed at 10 μL/min for 10 min to block any remaining active NHS. Competition assays were performed using NextStep injections in the OneStep assay builder. Pre- and post-immune MsrA/B mouse sera were used as the first injection (A), and Met(O) as the second injection (B), with PBS used as a negative control. The competition injection was run for 60 s with the A starting at a 1:100 dilution of serum at time zero and reducing across the injection time, with the B component increasing across the injection reaching 10 μM at 60 s. Binding of Met(O) to MsrA/B was compared with and without serum, and with pre- and post-immune serum. Data was collected using the Pioneer Software package and analyzed using Qdat analysis software. The percentage blocking was calculated based on the relative RMax of the Met(O) injection with and without serum, and the serum with and without Met(O).

## Results

### MsrA/B Is Highly Conserved in *N. gonorrhoeae* Strains

To investigate the distribution and conservation of MsrA/B in *N. gonorrhoeae* strains, a BLAST search was performed with MsrA/B from *N. gonorrhoeae* 1291 against available genomes. Analysis of *N. gonorrhoeae* genome strains in GenBank revealed that MsrA/B is highly conserved, being present in 100% of 468 strains, with 99–100% amino acid identity over the length of the 522 amino acid protein. An expanded search in the PubMLST database indicated that 86% of gonococcal strains (3,754/4,358) have an annotated *msrAB* gene [NEIS0020 (pilB/msrAB)]. This is likely an underestimate of the presence of *msrAB*, due to the presence of duplicate and incompletely annotated genomes. There are 35 unique gonococcal MsrA/B sequences in the PubMLST database, with 97.5–100% identity between them (Figure S1). There are four main variants that are present in 98% of strains, represented by strains PID322 (54% of strains), WHO_K (20%), 1291 (19%), and MS-11 (5%). The *N. gonorrhoeae* 1291 MsrA/B sequence is 98% identical to MsrA/B of *N. meningitidis* MC58 (Figure S1).

### MsrA/B Is a Surface Protein of the Pathogenic *Neisseria*

**Figure 1:**
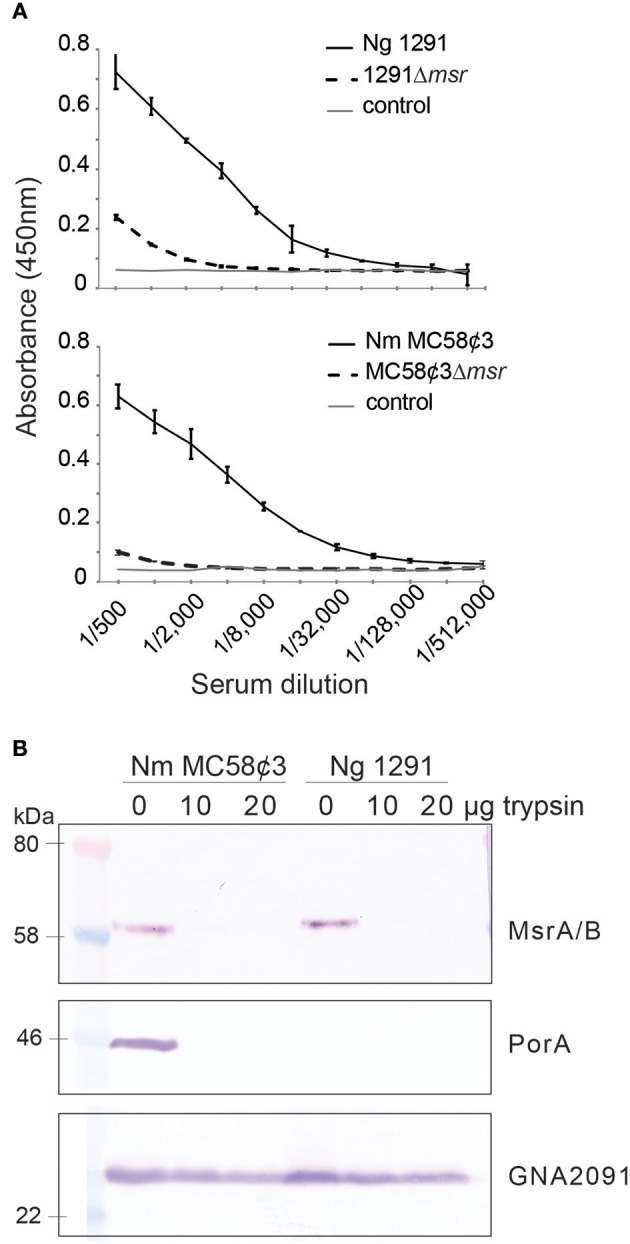
Surface localization of MsrA/B. **(A)** Whole cell ELISA of the wild type (WT) and *msr::kan* mutant (Δ*msr*) strains of *N. gonorrhoeae* 1291 and *N. meningitidis* MC58¢3, with anti-MsrA/B antibodies. The negative control containing secondary antibody only (control), is also shown. The graph shows the average absorbance at 450 nm from three independent replicates, ± one standard deviation. **(B)** Western blot analysis of trypsin treated (20 μg, 10 μg) and untreated (0 μg) whole cell *N. gonorrhoeae* 1291 and *N. meningitidis* MC58¢3, probed with antibodies to MsrA/B, the meningococcal surface protein PorA, and the intracellular protein GNA2091. No significant differences were seen in CFUs/ml at t0 vs. 60 min from samples taken at time 0 and 60 min (two-tailed unpaired Student's *t*-test *p* > 1.5), indicating that no cell lysis occurred during the assay (see Figure S3).

*N. gonorrhoeae* MsrA/B was proposed to be an outer membrane protein based on cell fractionation experiments (17), however the orientation of MsrA/B in the outer membrane was not determined. Our topology prediction analysis using TMHMM (39) indicates that MsrA/B does not have any transmembrane domains and that the whole protein is located outside of the membrane (Figure S2). To directly investigate if MsrA/B is surface exposed, whole cell ELISAs of *N. gonorrhoeae* 1291 and *N. meningitidis* MC58¢3 wild-type and *msr::kan* mutant strains were performed with mouse antisera raised against recombinant MsrA/B. Whole cell ELISA indicates that anti-MsrA/B bound to the wild-type 1291 and MC58¢3 intact cells (titer of 256,000 and 512,000, respectively), but binding was significantly reduced to the mutant strains (titer of 8,000 and 1,000, respectively; Figure 1A). In addition, MsrA/B was completely susceptible to digestion when intact bacterial cells were treated with 10 or 20 μg of trypsin for 60 min, similar to the meningococcal surface protein PorA (Figure 1B, Figure S3). The intracellular protein GNA2091 was not affected by trypsin treatment. This ELISA and Western data confirm that MsrA/B is on the surface of both *N. gonorrhoeae* and *N. meningitidis*. Trypsin treatment did not affect cell viability, as there was no significant difference in CFU counts between pre- and post-trypsin treatment (Figure S3).

### Immunogenicity of Recombinant Gonococcal MsrA/B

**Figure 2:**
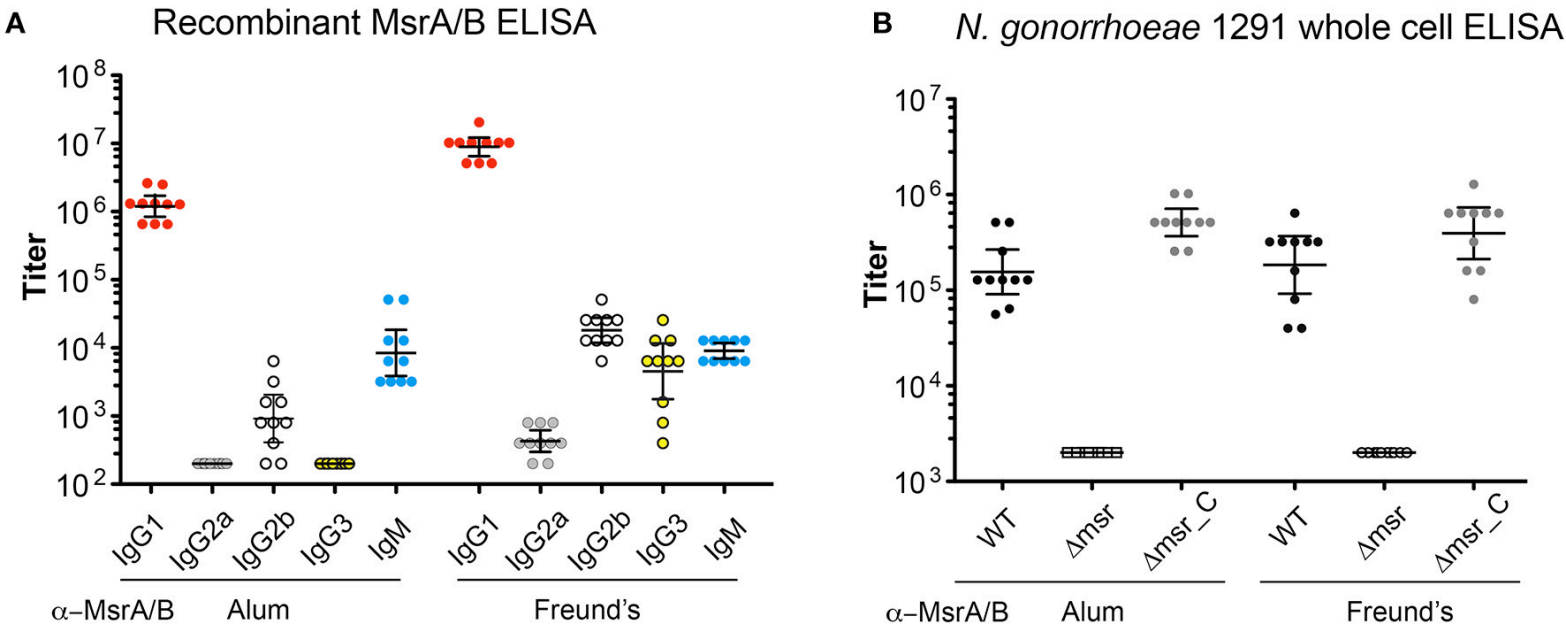
Immunogenicity of MsrA/B. The titers of the post-immune sera from each mouse immunized with either MsrA/B- Alum or MsrA/B-Freund's were determined by ELISA against **(A)** purified recombinant MsrA/B for IgG1, G2a, G2b, G3, IgM, or **(B)** whole cell *N. gonorrhoeae* 1291 wild type (WT), *msr::kan* mutant (Δmsr), and complemented (Δmsr_C) strains for IgG. The titer for each of 10 mice are shown with circles, and the geometric mean titer (GMT) and 95% Confidence interval are indicated bars. The titers of pre-immune sera against whole cell *N. gonorrhoeae* 1291 strains were ≤ 200.

To investigate the immunogenicity of MsrA/B, 10 mice were immunized with recombinant MsrA/B with either aluminum hydroxide (MsrA/B-Alum) or Freund's adjuvant (MsrA/B-Freund's). The sera were assessed by ELISA and Western blot. ELISA results with recombinant MsrA/B indicate a dominant IgG1 response in mice immunized with MsrA/B and either adjuvant, with a geometric mean titer (GMT) of 1,222,945 for MsrA/B-Alum and 8,914,438 for MsrA/B-Freund's (Figure 2A). Higher titers of IgG2a, IgG2b, and IgG3 were detected in mice immunized MsrA/B-Freund's compared to MsrA/B-Alum, while IgM titers were similar for both adjuvants (Figure 2A, Tables S2, S3).

**Figure 3:**
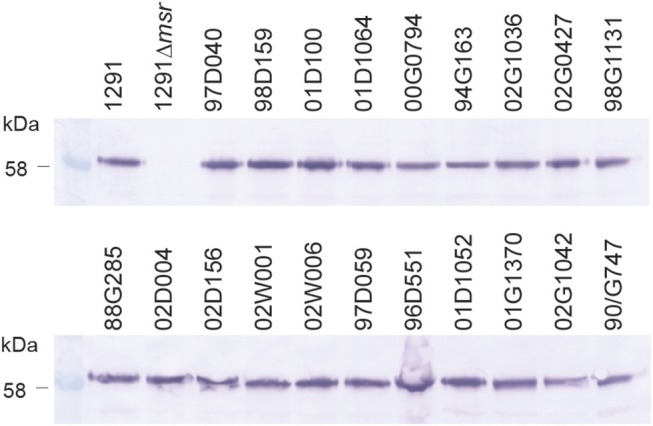
Expression of MsrA/B in a panel of gonococcal strains. Western blot analysis of MsrA/B expression in a panel of *N. gonorrhoeae* strains, including the 1291 wild type and *msr::kan* mutant (1291Δ*msr*), and 20 clinical isolates from mucosal and disseminated gonococcal infections.

Whole cell ELISA of the *N. gonorrhoeae* 1291 wild-type, 1291*msr::kan* mutant, and complemented strains indicated that the MsrA/B antisera from each mouse was able to recognize the native MsrA/B protein on the bacterial surface (Figure 2B, Tables S2, S3). There was a similar response against the wild-type from mice immunized with either adjuvant [GMT of 155,496 for MsrA/B-Alum, 183,792 for MsrA/B-Freund's (*p* = 0.52)] and a significantly reduced response to the *msr::kan* mutant strain (GMT of 2,000 for both adjuvant, *p* < 0.001 vs. wild-type). Analysis of MsrA/B-antisera by Western blotting against whole cell lysates of *N. gonorrhoeae* wild-type and the *msr::kan* mutant confirmed that MsrA/B antisera specifically recognize MsrA/B. There was no reactivity against MsrA/B in pre-immune sera, while an antibody response was generated by all mice that specifically recognizes MsrA/B in the wild-type strain (Figure S4). This ELISA and Western data confirm that MsrA/B is immunogenic and that anti-MsrA/B antisera can specifically recognize MsrA/B on the surface of *N. gonorrhoeae*. The expression of MsrA/B and the cross-reactivity of the MsrA/B antisera was confirmed by Western blot analysis of 20 clinical isolates from mucosal and disseminated gonococcal infections (Figure 3, Figure S5).

### MsrA/B Antisera Has Bactericidal and Opsonophagocytic Activity

**Figure 4:**
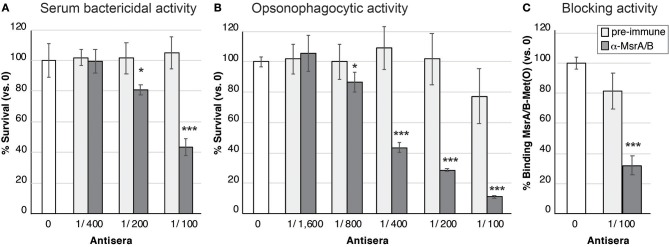
Functional activity of MsrA/B antisera against *N. gonorrhoeae*. **(A)** Serum bactericidal activity. The survival of the *N. gonorrhoeae* in the presence of two-fold dilutions of heat inactivated pre-immune (light gray) or α-MsrA/B (dark gray) sera, plus 10% normal human serum as a complement source is shown. **(B)** Opsonophagocytic activity. The survival of the *N. gonorrhoeae* in the presence of two-fold dilutions of heat inactivated pre-immune (light gray) or α-MsrA/B (dark gray) sera, plus primary human PMNs and 10% normal human serum as a complement source is shown. For **(A,B)**, data represent the mean survival (±1 standard deviation) for triplicate samples, as a percentage of bacteria in the absence of antibody (the no antibody control (white) set at 100%, represents 2.0 × 10^3^ CFU for SBA and 3.5 × 10^3^ CFU for OPA). **(C)** Blocking of MsrA/B binding to Met(O). Surface plasmon resonance (SPR) of MsrA/B interaction with Met(O) was performed in the presence of pre-immune (light gray) or α-MsrA/B (dark gray) sera. Data represents the mean MsrA/B-Met(O) binding (±1 standard deviation) for triplicate samples, as a percentage of MsrA/B-Met(O) binding in the absence of antibody (the no antibody control (white) set at 100%, represents a K_D_ of 15.4 ± 3.7 nM). For **(A–C)**, statistically significant differences relative to the no serum controls, using a two-tailed Student's *t*-test are indicated: *P < 0.05; ***P ≤ 0.001.

Mouse MsrA/B-Alum and MsrA/B-Freund's antisera were investigated for its ability to elicit serum bactericidal activity (SBA) and opsonophagocytic (OPA) killing of *N. gonorrhoeae*. Incubation of *N. gonorrhoeae* with serial dilutions of pooled MsrA/B antisera and human serum as the complement source, indicated that MsrA/B-Freund's antisera mediated dose-dependent killing, with an SBA titer of 100 (Figure 4A). SBA analysis of MsrA/B-Freund's sera from the 10 individual mice showed dose-dependent killing for 9/10 mice, and a ≥two-fold increase in SBA titer from pre- to post-immune sera for 8/10 mice (Tables S2, S3). Minimal killing was seen for the MsrA/B-Alum serum at the dilutions tested (titer < 50; Table S2).

Incubation of *N. gonorrhoeae* with pooled MsrA/B-Freund's antisera, human PMNs, and human serum as a complement source, revealed dose-dependent opsonophagocytic killing, with a titer of 400 (Figure 4B). Analysis of MsrA/B-Freund's serum from the 10 individual mice showed dose-dependent killing, and a ≥two-fold increase in OPA titer from pre- to post-immune sera for 9/10 mice (Tables S2, S3). The MsrA/B-Alum antisera did not mediate any opsonophagocytic killing (Tables S2, S3).

### MsrA/B Antisera Is Able to Block MsrA/B Binding to Met(O)

To investigate whether MsrA/B antisera was able to block the functional role of MsrA/B, SPR analysis of MsrA/B binding to methionine sulfoxide [Met(O)] was performed in the absence of serum, and in the presence of pre-immune and MsrA/B-Freund's antisera. Recombinant MsrA/B was immobilized on the SPR sensor chip and free Met(O) was flowed over the immobilized protein. MsrA/B bound to Met(O) with a high affinity, with a K_D_ (equilibrium dissociation constant) of 15.4 ± 3.7 nM (Figure S6). A competition assay with pooled MsrA/B-Freund's antisera reduced MsrA/B-Met(O) binding from 100 to 32% (Figure 4C; *p* ≤ 0.002 vs. no serum or pre-immune sera), while pooled pre-immune sera did not significantly reduce MsrA/B-Met(O) interactions (81 ± 12% binding, *p* = 0.05). Screening of the individual sera showed that 9/10 mice sera significantly blocked MsrA/B-Met(O) binding, compared to no serum and pre-immune serum controls (*p* < 0.05, Table S2), with serum from one mouse blocking >99% of MsrA/B binding to Met(O).

## Discussion

Antimicrobial resistant *N. gonorrhoeae* is currently an urgent threat to public health worldwide and development of a vaccine is the optimal long-term solution to control gonorrhea [reviewed in Edwards et al. (3)]. Targeting bacterial factors important for survival or virulence is a potential strategy for vaccine development, as a protective immune response against *N. gonorrhoeae* may encompass both functional blocking of human-specific disease processes as well as conventional immune killing processes. The gonococcal methionine sulfoxide reductase MsrA/B plays an important role in protecting *N. gonorrhoeae* from oxidative damage (17), by catalyzing the reduction of methionine sulfoxide residues Met(O) back to methionine (Met) (14, 20). Therefore, we investigated its potential as a gonococcal vaccine antigen. Our results showed that MsrA/B is highly conserved and surface exposed in *N. gonorrhoeae*. Furthermore, we demonstrated that antisera raised to recombinant MsrA/B mediates bactericidal and opsonophagocytic killing of *N. gonorrhoeae* and is able to inhibit binding of MsrA/B to its substrate, methionine sulfoxide [Met(O)].

The majority of bacterial methionine sulfoxide reductase systems consist of separate cytoplasmic MsrA and MsrB proteins, which are specific for the Met-S(O) and Met-R(O) epimers, respectively. During the catalytic process, firstly a sulfenic intermediate is produced with concurrent release of the repaired Met and, secondly, a recycling step occurs where oxidized MsrA and/or MsrB are reduced to their active form via a thioredoxin/thioredoxin reductase system (29). However, in *N. gonorrhoeae* the MsrA, MsrB and thioredoxin enzymatic functions are present in a single protein, MsrA/B, that is located in the outer membrane (17). MsrA/B was presumed to be facing the periplasmic space, however, we have determined that the gonococcal and meningococcal MsrA/B protein is on the surface. To our knowledge, this is the first time that a Msr has been reported to be surface exposed in a Gram-negative bacterium. In other examples of Gram-negative methionine sulfoxide reductases, the enzyme exists in the cytoplasm and utilizes the cytoplasmic thiol pool regenerated by thioredoxin reductase. It is not clear how MsrA/B of *N. gonorrhoeae* exchanges thiols to maintain activity when it is on the outside of the bacterial cell. A similar problem exists in understanding electron transport with respect to the outer membrane located nitrate reductase AniA, which is active but has no obvious electron donor (40). More work is required to understand how these enzymes function in the context of their localization.

MsrA/B is highly conserved in *N. gonorrhoeae*, with >97% amino acid identity in all strains investigated. Overall, only two sites had common variations: Thr31 substitution to Ala31 in ~75% of isolates, and Lys520 substitution to Glu520 in ~25% of isolates. Residue 31 is in the predicted signal peptide of MsrA/B, and all other variant amino acid residues are not located in any known catalytic domains identified in *N. meningitidis* MsrA/B (25, 26, 41). According to Skaar et al. (17), a truncated version of Msr was observed in the cytoplasm and this might due to the miss-cleavage of the signal peptidase. However, we did not see any evidence of a truncated version of MsrA/B in our analysis and believe that the majority of protein is surface exposed. This is not the first example of a gonococcal protein that was found to be surface exposed, despite previously being considered to be a periplasmic protein. For example, both AniA (40) and MetQ (38) are surface exposed on *N. gonorrhoeae*. The AniA nitrite reductase is important for the growth and survival of *N. gonorrhoeae* under oxygen-limited conditions (42) and for biofilm formation (43). Antibodies raised to AniA are capable of functional blocking, able to block AniA nitrite reductase activity of *N. gonorrhoeae* (40). Similarly, MetQ, the methionine binding component of an ABC transporter system is involved in gonococcal adherence to cervical epithelial cells, and antibodies raised to MetQ are able to block gonococcal adherence to epithelial cells and mediate bactericidal activity (38).

MsrA/B was immunogenic in mice when presented with either Alum or Freund's as an adjuvant and antibodies were able to recognize MsrA/B on the surface of *N. gonorrhoeae* via whole cell ELISA. MsrA/B immunization with either Alum and Freund's induced a predominantly IgG1 immune response. Antibodies raised to MsrA/B-Freund's, but not MsrA/B-Alum, mediated killing of *N. gonorrhoeae* via both serum bactericidal activity and opsonophagocytic activity. This activity is probably due to the induction of IgG3 as well as IgG2a and IgG2b by the Freund's adjuvanted MsrA/B antigen. This is consistent with previous findings that mouse IgG isotypes vary in their capacity to activate complement; for example, mouse antibodies targeting PorA of *N. meningitidis* have a hierarchy of IgG3>IgG2>IgG1 for both serum bactericidal and opsonophagocytic activity (44). Anti-MsrA/B-Freund's was also able to block the activity of MsrA/B by inhibiting binding to its substrate. In summary, based on the conservation and immunogenicity of MsrA/B, as well as the functional activity of antibodies raised to it, we propose MsrA/B as a vaccine antigen for *N. gonorrhoeae*.

## Author Contributions

FJ, ES, and CD performed the experiments. FJ, ES, CD, and KS contributed to writing the manuscript. MJ and KS conceived of the study, directed the collection and analysis of experimental data, edited the manuscript.

### Conflict of Interest Statement

The authors declare that the research was conducted in the absence of any commercial or financial relationships that could be construed as a potential conflict of interest.
