# Supplementary material for: The Neisseria gonorrhoeae Methionine Sulfoxide Reductase (MsrA/B) Is a Surface Exposed, Immunogenic, Vaccine Candidate
Source: Front Immunol. 2019 Feb 6;10:137. doi: 10.3389/fimmu.2019.00137 (PMC6372556; doi:10.3389/fimmu.2019.00137)
Supplement: Supplementary file 1 [file Data_Sheet_1.PDF]

The *Neisseria gonorrhoeae* methionine sulfoxide reductase (MsrA/B) is a surface exposed, immunogenic, vaccine candidate

Freda E.-C. Jen[1], Evgeny A. Semchenko[1], Christopher J. Day[1], and Kate L. Seib[1§], Michael P. Jennings[1§*]

[1]Institute for Glycomics, Griffith University, Gold Coast, QLD, Australia

Supplementary data

Table S1. Primers used in this study.

Primer name	Primer sequence 5'-3' *
1291msrFor	GCCGTCTGAAATGAAACACCGTACTTTCTTTTCCC
1291msrRev	TTCAGACGGCTTATTTCACTTTGCCCTTCAACGCG
msrexp_NdeIF	AAAATCCATATGAAAGGGACCGCGACCGTGCCGCA
msrexp_XhoIR	CCCTGACTCGAGTTATTTCACTTTGCCCTTC
pCTS32_Msr_AflIIFor	CTCGAGCTTAAGCCGGCGTTTCCTGTTTTTC
pCTS32_Msr_SmaIRev	TGCGGCCCCGGGTTATTTCACTTTGCCCTTCAACG
pComP_Msr_NdeIFor	ATCAAACATATGATGAAACACCGTACTTTCTTTTCC
pComP_Msr_NsiIRev	TGCGGCATGCATTTATTTCACTTTGCCCTTCAACG

Table S2. Data for individual and pooled mice sera immunised with MsrA/B-Alum.

MsrA/B-Alum	ELISA titre vs MsrA/B					ELISA titre vs whole cells^			SBA titre		OPA titre	
**Mouse**	**IgG1**	**IgG2a**	**IgG2b**	**IgG3**	**IgM**	**WT**	**Δmsr**	**Δmsr_C**	**Pre**	**Post**	**Pre**	**Post**
**1**	655,360	200	800	200	12,800	128,000	2,000	512,000	-	-	-	-
**2**	2,621,440	200	3,200	200	51,200	256,000	2,000	512,000	-	-	-	-
**3**	1,310,720	200	800	200	6,400	64,000	2,000	256,000	-	-	-	-
**4**	1,310,720	200	1,600	200	6,400	512,000	2,000	1,024,000	-	-	-	-
**5**	655,360	200	1,600	200	3,200	56,000	2,000	512,000	-	-	-	-
**6**	1,310,720	200	200	200	3,200	128,000	2,000	512,000	-	-	-	-
**7**	1,310,720	200	800	200	3,200	128,000	2,000	256,000	-	-	-	-
**8**	1,310,720	200	6,400	200	12,800	512,000	2,000	1,024,000	-	-	-	-
**9**	655,360	200	400	200	51,200	128,000	2,000	512,000	-	-	-	-
**10**	2,621,440	200	200	200	3,200	128,000	2,000	512,000	-	-	-	-
**GMT**	1,222,945	200	919	200	8,445	155,496	2,000	512,000	-	-	-	-
**pool**									<50	<50	<50	<50

SBA titre; serum bactericidal titre (reciprocal of the lowest antibody dilution which induced more than 50% killing after 60 min). OPA titre, opsonophagocytic titre (reciprocal of the lowest antibody dilution which induced more than 50% killing after 90 min). GMT, geometric mean titre; -, not determined. ^ The titres of pre-immune sera against whole cell *N. gonorrhoeae* 1291strains were ≤200.

Table S3. Data for individual and pooled mice sera immunised with MsrA/B-Freund's.

MsrA/B-Freund's	ELISA titre vs MsrA/B					ELISA titre vs whole cells^			SBA titre*		OPA titre*		MsrA/B-Me(O) binding inhibition	
**Mouse**	**IgG1**	**IgG2a**	**IgG2b**	**IgG3**	**IgM**	**WT**	**Δmsr**	**Δmsr_C**	**Pre**	**Post**	**Pre**	**Post**	**Pre**	**Post**
**1**	10,240,000	400	51,200	25,600	12,800	320,000	2,000	640,000	<50	50	<100	200	20.5%	41.9%
**2**	5,120,000	800	25,600	6,400	12,800	320,000	2,000	640,000	<50	50	100	200	4.6%	62.5%
**3**	10,240,000	400	25,600	6,400	6,400	320,000	2,000	640,000	<50	100	<100	200	5.7%	99.7%
**4**	10,240,000	800	25,600	12,800	12,800	320,000	2,000	640,000	<50	50	<100	200	9.0%	65.7%
**5**	10,240,000	800	12,800	6,400	6,400	80,000	2,000	160,000	<50	<50	<100	<100	6.2%	12.0%
**6**	20,480,000	400	12,800	1,600	6,400	40,000	2,000	160,000	50	200	100	800	3.2%	49.0%
**7**	10,240,000	200	12,800	6,400	12,800	160,000	2,000	320,000	<50	<50	<100	100	1.1%	54.1%
**8**	10,240,000	400	25,600	12,800	6,400	40,000	2,000	80,000	50	100	100	400	6.5%	54.7%
**9**	5,120,000	200	12,800	800	12,800	640,000	2,000	1,280,000	50	200	100	400	17.2%	64.9%
**10**	5,120,000	400	6,400	400	6,400	320,000	2,000	640,000	<50	50	<100	100	0.9%	55.9%
**GMT**	8914438	429	18102	4525	9051	246754	2000	393966	50	84	55	200		
**Mean**													7.5%	56.0%
**pool**									<100	100	<100	400	18.5%	67.9%

SBA titre; serum bactericidal titre (reciprocal of the lowest antibody dilution which induced more than 50% killing after 60 min). OPA titre, opsonophagocytic titre (reciprocal of the lowest antibody dilution which induced more than 50% killing after 90 min). GMT, geometric mean titre. ^ The titres of pre-immune sera against whole cell *N. gonorrhoeae* 1291strains was ≤200. * When a final titre was not reached (i.e., <50 or <100) a value of the next 2-fold dilution (i.e., 25 or 50, respectively) was used to calculate the GMT.

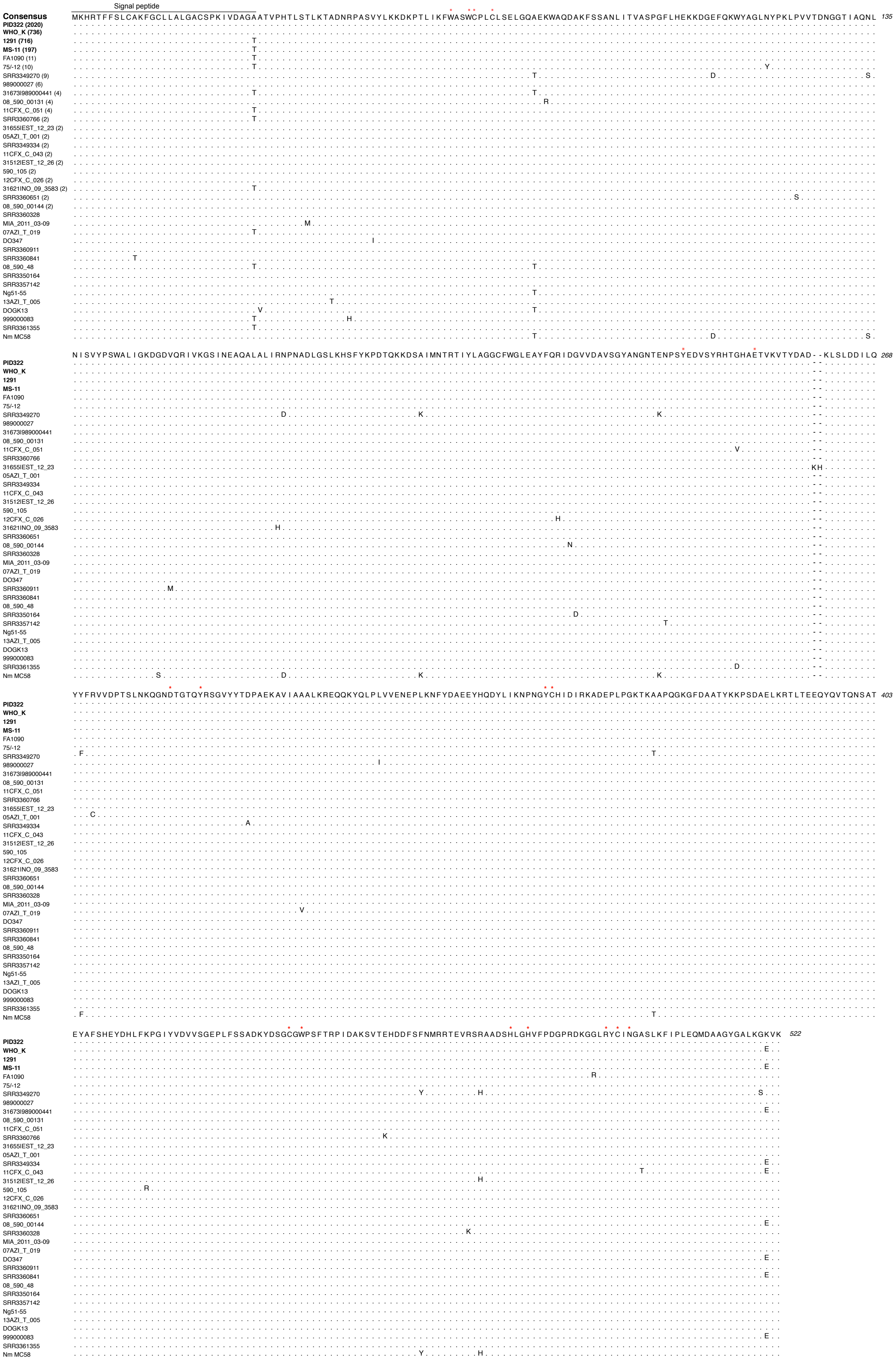
Signal peptide
Consensus
MKHRTFFSLCAKFGCLLALGACSPKIVDAGAATVPHTLSTLKTADNRPASVYLKKDKPTLIKFWASWCPLCLSELGQAEKWAQDAKFSSANLITVASPGFLHEKKDGEFQKWYAGLNYPKLPVVTDNGGTIAQNL 135
NISVYPSWALIGKDGDVQRIVKGSINEAQALALIRNPNADLGSLKHSFYKPDTQKKDSAIMNTRTIYLAGGCFWGLEAYFQRIDGVVDAVSGYANGNTENPSYEDVSYRHTGHAETVKVTYDAD--KLSLDDILQ 268
YYFRVVDPTSLNKQGNDTGTQYRSGVYYTDPAEKAVIAAALKREQQKYQLPLVVENEPLKNFYDAEEYHQDYLIKNPNGYCHIDIRKADEPLPGKTKAAPQGKGFDAATYKKPSDAELKRTLTEEQYQVTQNSAT 403
EYAFSHEYDHLFKPGIYVDVVSGEPLFSSADKYDSGCGWPSFTRPIDAKSVTEHDDFSFNMRRTEVRSRAADSHLGHVFPDGPRDKGGLRYCINGASLKFIPLEQMDAAGYGALKGKVK 522
PID322 (2020)
WHO_K (736)
1291 (716)
MS-11 (197)
FA1090 (11)
75/-12 (10)
SRR3349270 (9)
989000027 (6)
31673l989000441 (4)
08_590_00131 (4)
11CFX_C_051 (4)
SRR3360766 (2)
31655lEST_12_23 (2)
05AZI_T_001 (2)
SRR3349334 (2)
11CFX_C_043 (2)
31512lEST_12_26 (2)
590_105 (2)
12CFX_C_026 (2)
31621lNO_09_3583 (2)
SRR3360651 (2)
08_590_00144 (2)
SRR3360328
MIA_2011_03-09
07AZI_T_019
DO347
SRR3360911
SRR3360841
08_590_48
SRR3350164
SRR3357142
Ng51-55
13AZI_T_005
DOGK13
999000083
SRR3361355
Nm MC58

Figure S1. Conservation of gonococcal MsrA/B.

An alignment of the 35 unique MsrA/B variants of *N. gonorrhoeae* is shown, with the consensus sequence at the top. Matches to the consensus sequence are indicated by dots. A representative strain for each variant is shown and the number of strains with that variant is given in brackets. The *N. meningitidis* MC58 MsrA/B sequence is also included. The predicted signal peptide is shown, and * indicate catalytic residues identified in *N. meningitidis* MsrA/B.

TMHMM result

HELP with output formats

```
# Ng1291_MsrA/B Length: 522
# Ng1291_MsrA/B Number of predicted TMHs:  0
# Ng1291_MsrA/B Exp number of AAs in TMHs: 0.651070000000000001
# Ng1291_MsrA/B Exp number, first 60 AAs:  0.61189
# Ng1291_MsrA/B Total prob of N-in:        0.03201
Ng1291_MsrA/B   TMHMM2.0        outside      1   522
```

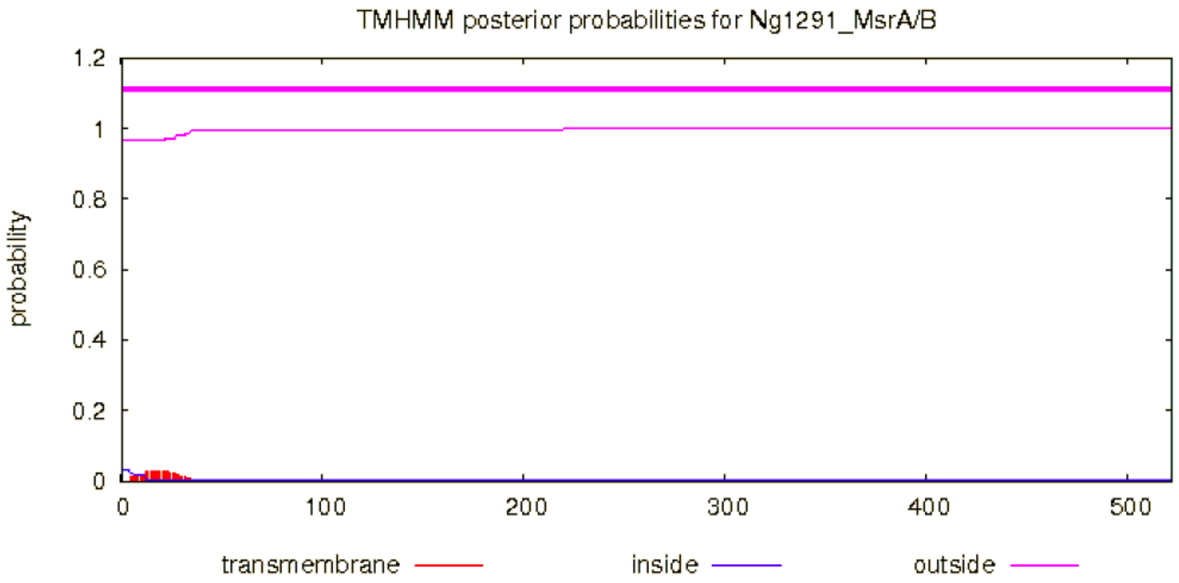

plot in postscript, script for making the plot in gnuplot, data for plot

Figure S2. Topology prediction of MsrA/B.
Topology prediction analysis using TMHMM (Krogh et al., 2001) indicates that MsrA/B does not have any transmembrane domains and that the whole protein is located outside of the membrane.

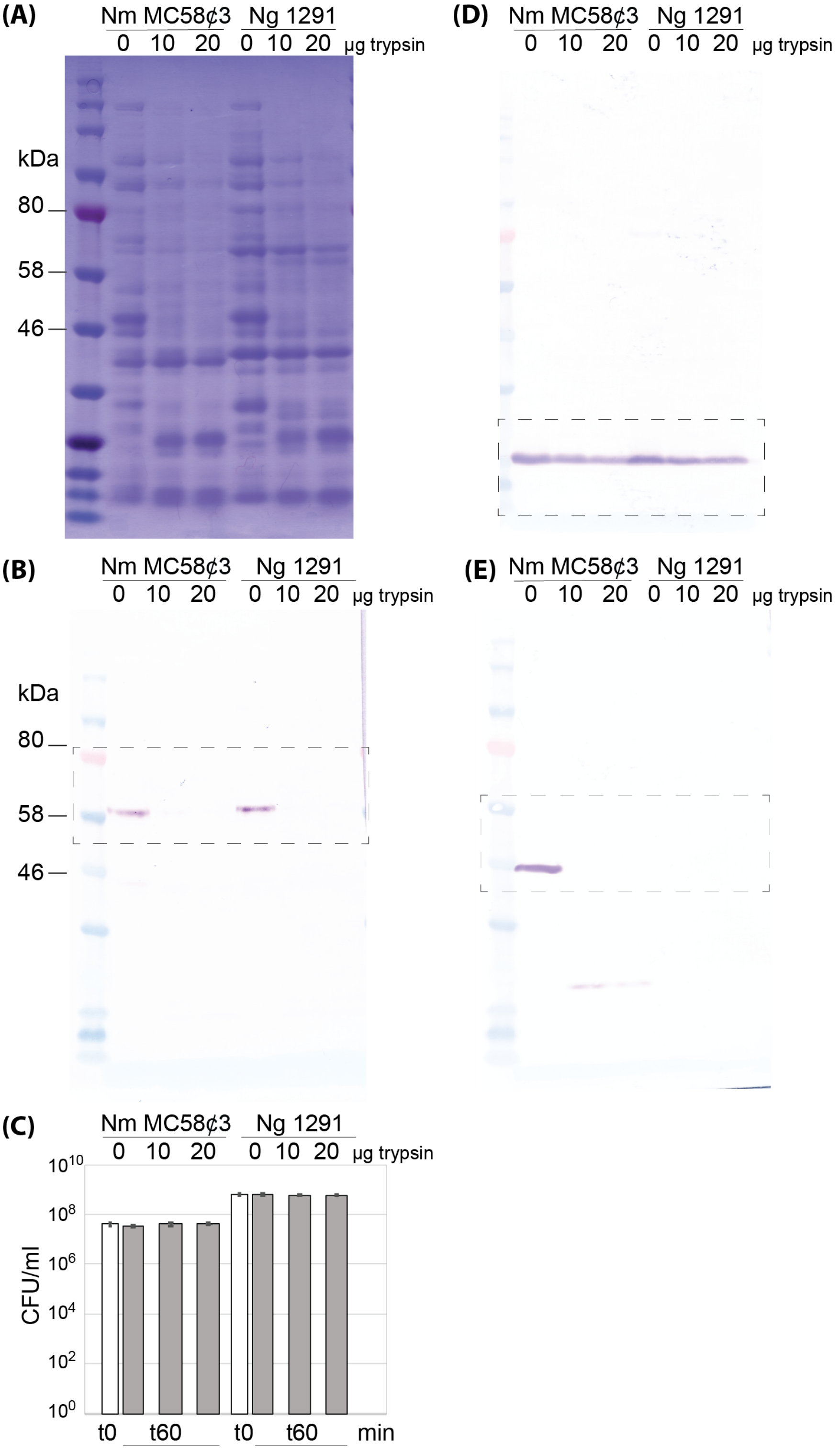

Figure S3. Surface localisation of MsrA/B.

(A) Coomassie stained SDS-PAGE and **(B)** Western blot analysis of trypsin treated (20 µg, 10µg) and untreated (0 µg) whole cell *N. gonorrhoeae* 1291 and *N. meningitidis* MC58¢3, probed with anti-MsrA/B antibodies. The region of the Western blot shown in Figure 1 is boxed. **(C)** CFU/ml determined from samples taken at time 0 (t0) and at 60 min (t60) for each sample are shown below the Western blot. No significant differences were seen in CFUs/ml at t0 vs. 60mins, as assessed by a two-tailed unpaired Student's *t-test* ($p > 1.5$), indicating that no cell lysis occurred during the assay. **(D-E)** Western blot analysis of samples from (A-C), probed with **(D)** antibodies to the intracellular protein GNA2091, and **(E)** antibodies to the extracellular protein PorA.

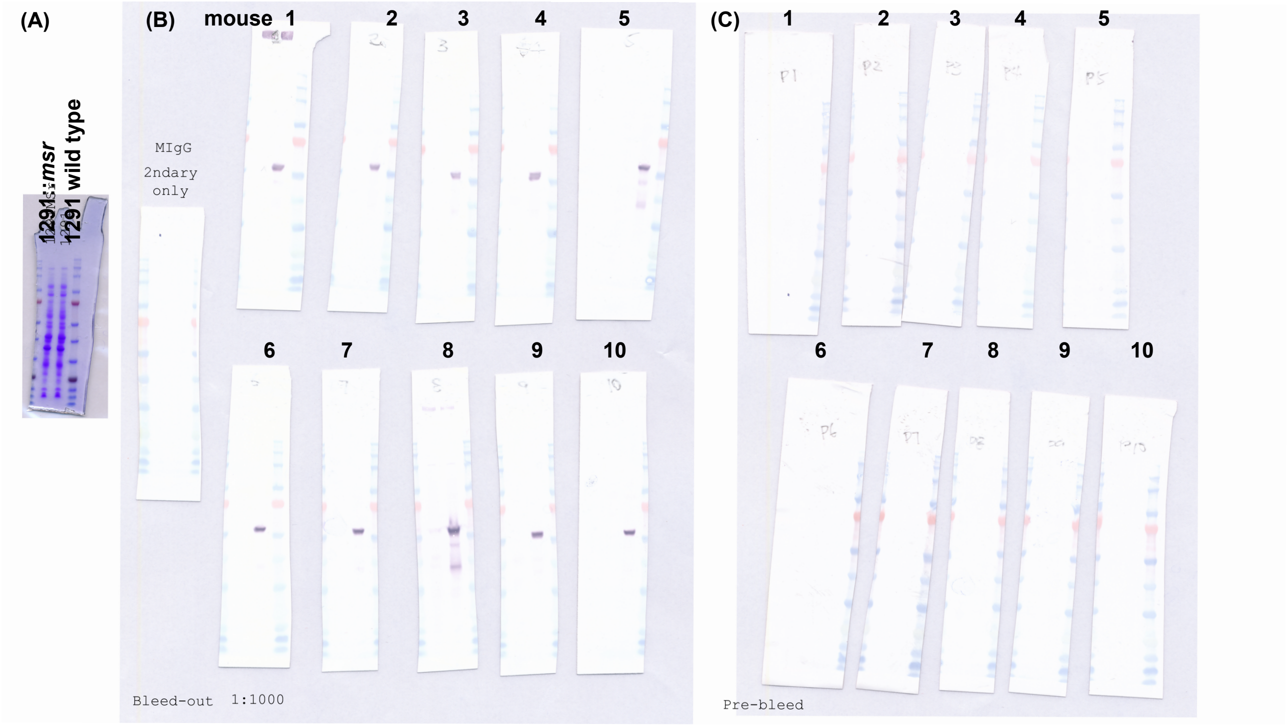

Figure S4. Reactivity of mouse sera to MsrA/B.

(A) Coomassie stained SDS-PAGE of *N. gonorrhoeae* 1291::*msr* and wild type whole cell lysates, and Western blot analysis of whole cell lysates probed with **(B)** anti-MsrA/B antibodies or **(C)** pre-immune sera from ten mice. There was no reactivity against MsrA/B in pre-immune sera, while an antibody response was generated by all mice that specifically recognizes MsrA/B in the wild-type strain but not the mutant strain.

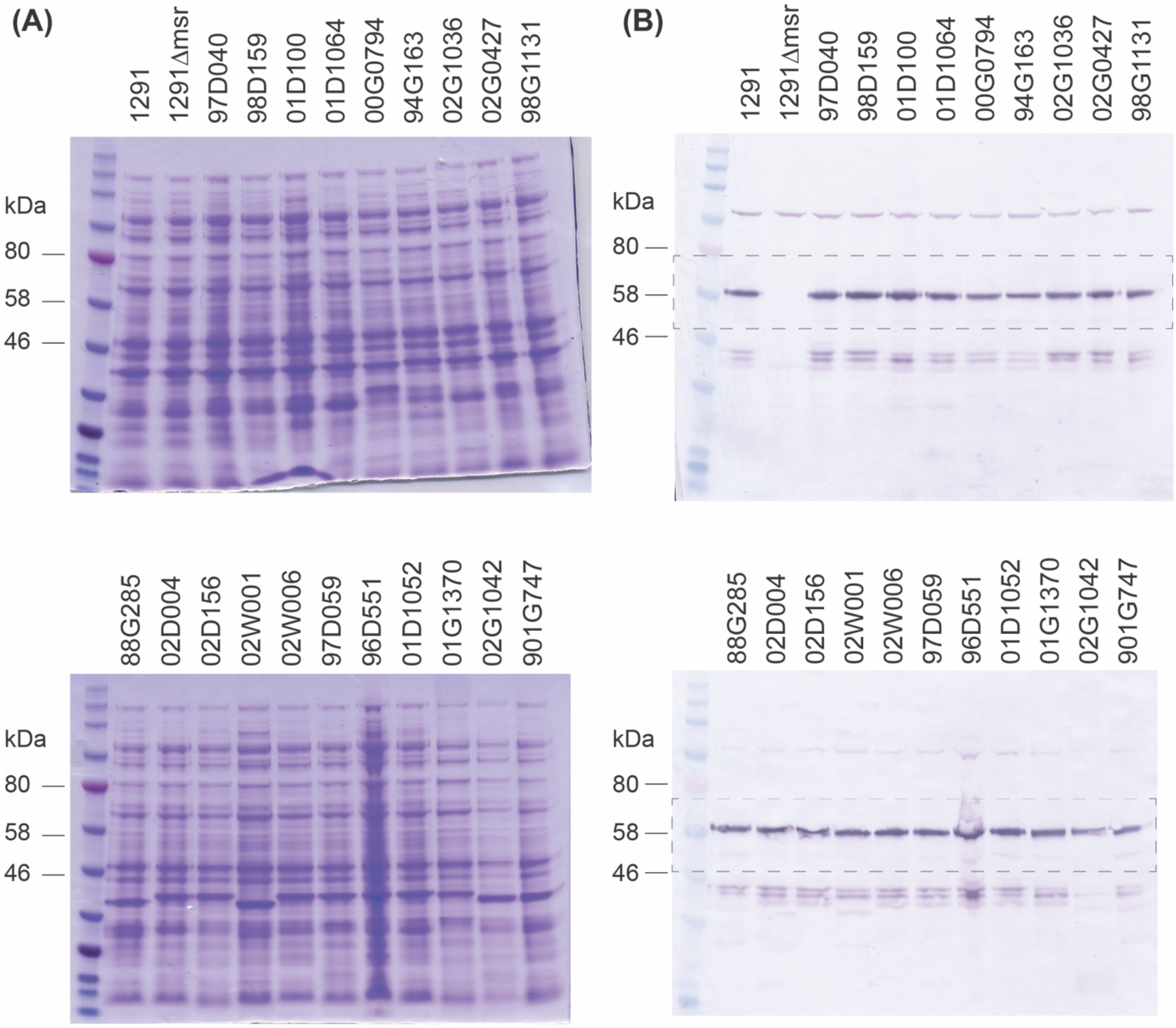

Figure S5. Expression of MsrA/B in a panel of gonococcal strains.
(A) Coomassie stains SDS-PAGE and (B) Western blot analysis of MsrA/B expression in a panel of *N. gonorrhoeae* strains, including the 1291 wild type and *msr::kan* mutant (1291Δ*msr*), and twenty clinical isolates from mucosal and disseminated gonococcal infections. The region of the Western blot shown in Figure 3 is boxed.

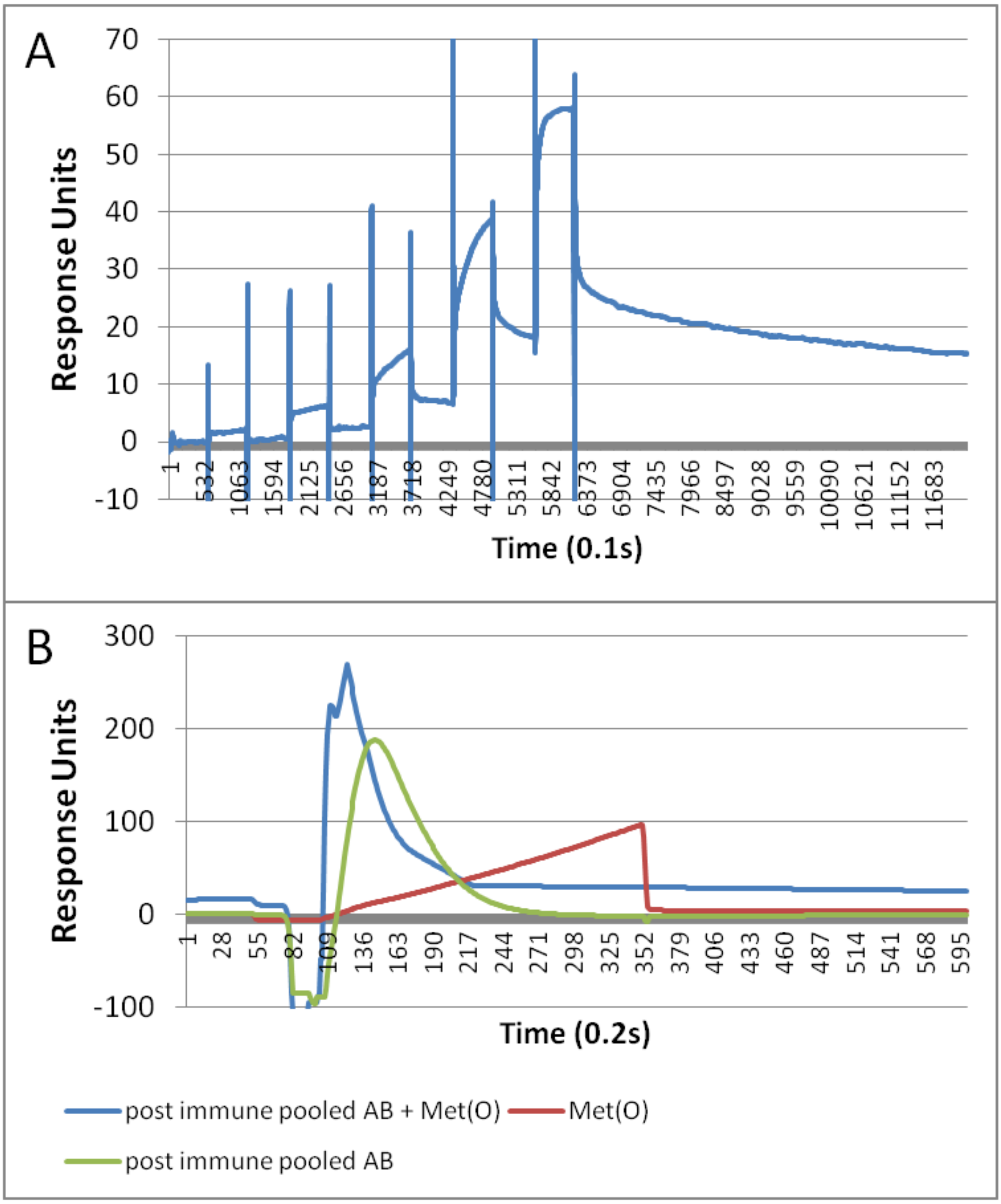

Figure S6. Surface plasmon resonance (SPR) analysis of MsrA/B - Met(O) interaction. Representative sensorgrams from surface plasmon resonance analysis of recombinant MsrA/B binding to methionine sulfoxide (Met(O)). **(A)** Single cycle kinetics of Met(O) against MsrA/B. **(B)** Competition analysis of MsrA/B with pooled post immune serum (AB) demonstrating the direct competition between the pooled serum and Met(O).
